# Mineral Composition of Organically Grown Wheat Genotypes: Contribution to Daily Minerals Intake

**DOI:** 10.3390/ijerph7093442

**Published:** 2010-09-06

**Authors:** Abrar Hussain, Hans Larsson, Ramune Kuktaite, Eva Johansson

**Affiliations:** Department of Agriculture–Farming Systems, Technology and Product Quality, Faculty of Landscape Planning, Horticulture and Agriculture Science, The Swedish University of Agricultural Sciences, Box 104, SE-23053 Alnarp, Sweden; E-Mails: Hans.Larsson@ltj.slu.se (H.L.); Ramune.Kuktaite@ltj.slu.se (R.K.); Eva.Johansson@ltj.slu.se (E.J.)

**Keywords:** organic genotypes, minerals, locations, spring and winter wheat, concentrations, food requirements

## Abstract

In this study, 321 winter and spring wheat genotypes were analysed for twelve nutritionally important minerals (B, Cu, Fe, Se, Mg, Zn, Ca, Mn, Mo, P, S and K). Some of the genotypes used were from multiple locations and years, resulting in a total number of 493 samples. Investigated genotypes were divided into six genotype groups *i.e.*, selections, old landraces, primitive wheat, spelt, old cultivars and cultivars. For some of the investigated minerals higher concentrations were observed in selections, primitive wheat, and old cultivars as compared to more modern wheat material, e.g., cultivars and spelt wheat. Location was found to have a significant effect on mineral concentration for all genotype groups, although for primitive wheat, genotype had a higher impact than location. Spring wheat was observed to have significantly higher values for B, Cu, Fe, Zn, Ca, S and K as compared to winter wheat. Higher levels of several minerals were observed in the present study, as compared to previous studies carried out in inorganic systems, indicating that organic conditions with suitable genotypes may enhance mineral concentration in wheat grain. This study also showed that a very high mineral concentration, close to daily requirements, can be produced by growing specific primitive wheat genotypes in an organic farming system. Thus, by selecting genotypes for further breeding, nutritional value of the wheat flour for human consumption can be improved.

## 1. Introduction

Minerals are important components required by humans in their daily food. Minerals are divided into two groups: (i) macro minerals, which are needed in large amounts, e.g., calcium (Ca), magnesium (Mg) and potassium (K), *etc.*, and (ii) micro minerals or trace elements, which are required in smaller quantities, e.g., copper (Cu), zinc (Zn), iron (Fe), boron (B), selenium (Se) [1]. All body processes depend upon the action of minerals to activate enzymes performing metabolic functions. Deficiency of specific minerals may lead to various chronic diseases [2,3]. Due to the high consumption of wheat in a variety of food products all over the world, wheat is considered an important source of minerals [4]. The concentration of minerals in wheat flour is genetically determined by the choice of cultivar and environmentally determined by soil, climate and management practices [5]. Concentration of minerals in wheat products can be increased by using whole grain flour instead of white flour. Also, suitable conditions together with selection of wheat cultivars that have a high mineral concentration, can be used to promote high mineral bioavailability [6]. Mineral concentration variability in cultivars has been studied by several authors using wheat grown in inorganic systems [5,7–12]. New techniques present today, allow investigations of a much higher number of minerals, as well as better accuracy as compared to the majority of the above mentioned studies.

Organic and inorganic growing conditions have been shown to have a great impact on the mineral concentrations of wheat grain [13–15]. For example, the concentrations of P and Mg were significantly higher in wheat varieties grown under inorganic conditions as compared with those produced organically [16]. The area under organic wheat production represents 18% of total arable organic land in Europe [17]. Reasons stated for increasing the area under organic production are: environmental concerns, increased nutritional quality and increased safety. Therefore, farmers are getting financial incentives from governments to convert the farming system from inorganic to organic [18].

In the present Western world society there is an increasing demand for and interest in high nutritional products. Organically grown food is seen by many as a nutritionally better solution as compared to conventionally produced food [19].

Primitive wheats such as Einkorn (*Triticum monococcum*) and Emmer (*T. dicoccum*) have been found to have high nutritional values in various aspects [20]. For example Einkorn has a high level of carotenoids and high protein concentration, compared to modern wheat [20]. Primitive wheat is the earliest cultivated form of wheat in organic farming system [21]. Thus, organically grown primitive species might be an optimal way of producing nutritional high value food. To our knowledge, there have been no investigations in which variation in mineral concentration among cultivars of different genetic background under organic cultivation has been studied.

The aim of this work was to screen wheat genotypes for mineral concentration under organic cultivation system conditions and to evaluate opportunities to produce wheat with exceptional high concentrations of minerals. The objective was also to evaluate differences in mineral composition between common and primitive wheats. Finally, another aim was to study location effects on mineral concentration.

## 2. Material and Methods

### 2.1. Sample Collection and Preparation

Wheat genotypes were collected mainly from the Nordic Gene Bank. Three hundred and twenty one winter and spring wheat genotypes were used for this study (Supplementary Table 1). These genotypes were grown organically during 2001–2007 at four locations in Sweden: Alnarp (55°39.4 N, 13°5.2 E), Bohuslän (58°31.6 N, 11°34.8 E), Gotland (57°36.6 N, 18°27.9 E) and Uppsala (59°49 N, 17°46.6 E). The soil type and characteristics are described in Table 1. Some of the genotypes used in the present study were therefore from multiple locations and years, resulting in a total number of 493 samples being available for analysis. The investigated genotypes could be divided into six genotype groups, *i.e.*, selections (n = 32), comprising wheat that has been selected for the purpose of organic cultivation from old material (spelt, durum and bread wheat breeding lines of), old landraces (n = 107), primitive wheat (n = 32) characterised by Einkorn (*Triticum monococcum*) and Emmer (*T. dicoccum),* spelt wheat (n = 103), old cultivars (n = 191) from 1900–1960, cultivars (n = 28) after 1970 (Supplementary Table 1). Plant height was measured from soil surface to tip of the spike, awns not included. Yield data was calculated for 28 genotypes which were grown in bigger plots in Bohuslän.

About 40 g of each grain sample was milled to flour (whole grain) for 1 min with a laboratory mill (Yellow line, A10, IKA-Werke, Staufen, Germany). Afterward the flour samples were stored at −20 °C.

### 2.2. Sample Drying and Digestion

All the samples were dried in an oven at 40 °C for 24 h and 0.5 g of each flour sample was measured and digested with microwave digester (microwave labstation Mars 5, CEM Corporation, Mathews, NC, USA) using 10 mL of concentrated nitric acid (HNO_3_). The digested samples were diluted with water to 100 mL before analysis.

### 2.3. Chemical Analysis

The mineral concentration in the solutions was measured by using Inductively Coupled Plasma Mass Spectrometry (ICP-MS; Perkin-Elmer, ELAN-6000) and by Inductively Coupled Plasma Atomic Emission Spectrometry (ICP-OES; Perkin-Elmer, OPTIMA 3000 DV). Standards used in the analysis were atomic spectrometry standards from Perkin-Elmer, SPEX, AccuStandard and Merck.

Calibration of the ICP-OES instrument was done by using a mixed multicomponent standard at three concentrations within the factor of 50 and calibration was maintained with independent standards. The ICP-MS instrument was calibrated against four mixed standards. Among these four, three were prepared from a single element standard and one was the Merck’s ICP multi element standard IV. Rhodium was added to all samples and standard solutions as an internal standard.

Mineral elements Al, B, Ba, Ca, Cu, Fe, K, Mg, Mn, Na, P, S, Sr, Ti and Zn were determined by ICP-OES. Other elements measured by ICP-MS (isotopes used indicated) were ^75^As, ^59^Co, ^52^Cr, ^202^Hg, ^7^Li, ^95^Mo, ^58^Ni, ^208^Pb, ^121^Sb, ^82^Se, ^120^Sn, ^51^V. Blanks were treated identically and together with samples. Detection limits of minerals determined, expressed as three time the standard deviation (3 × S.D.) and calculated for multiple determination of the blanks treated as the samples, were reported by [22]. All analyses were performed at the ICP laboratory, Department of Ecology, Lund University, which has 30 year experience of mineral analysis and participate in regular national and international multi-element calibration tests, always with very good results. The concentration for each element was calculated as absolute concentration mg/kg.

### 2.4. Statistical Analysis

Analysis of Variance (ANOVA), Spearman rank correlation and variances were calculated by using Statistical Analysis System (SAS Institute, Cary, NC 1985). The ratio between genetic group and location variances (σ_g_^2^/σ_l_^2^) was calculated for each of the analysed minerals and genotype groups. This ratio provides a mean for determining relative influences of genotype and location on the mineral composition of wheat. A ratio more than 1.0 indicates greater influence of genotype on variability of minerals and less than 1.0 ratio shows a greater impact of location [9].

## 3. Results

The concentration of 27 minerals was determined in the wheat flour samples and in the present paper variation in the twelve minerals (B, Cu, Fe, Se, Mg, Zn, Ca, Mn, Mo, P, S and K) that are essential for good preventive nutrition of human are evaluated and discussed.

### 3.1. Genotypic Differences

Substantial differences in the concentration of grain minerals were found among genotype and genotype groups (Table 2). Selections showed significantly the highest concentration of Se (0.18 mg/kg) among the genotype groups. High concentrations of Fe (35.8 mg/kg), Mg (1330 mg/kg), Mn (23.7 mg/kg), Mo (2.58 mg/kg), P (4,670 mg/kg) and S (1,310 mg/kg) were also found in the selections as compared to several of the other genotype groups (Table 2).

Old cultivars were found to have high concentrations of Fe (39.4 mg/kg), Ca (390 mg/kg) and Mn (24.2 mg/kg) as compared to several other genotype groups. Concentrations of B (1.90 mg/kg), Se (0.10 mg/kg), Mg (1,220 mg/kg), Zn (38.1 mg/kg), Mo (1.53 mg/kg), P (3,890 mg/kg) and K (3,980 mg/kg) were observed to be relatively low in old cultivars among the genotype groups (Table 2).

Primitive wheat showed significantly the highest concentrations of Zn (45.6 mg/kg) and K (4,670 mg/kg) compared to all other genotype groups. Grain concentrations of B (2.41 mg/kg), Cu (5.75 mg/kg), Mg (1,300 mg/kg), Ca (383 mg/kg), P (4,540 mg/kg) and S (1,350 mg/kg) were also observed to be high in primitive wheat compared to the rest of the genotype groups. Primitive wheat revealed relatively low magnitude for Se (0.11 mg/kg), Fe (32.2 mg/kg), Mn (17.6 mg/kg) and Mo (1.36 mg/kg) compared to the other genotype groups (Table 2).

Spelt wheat showed high concentrations of Cu (5.50 mg/kg), Fe (38.0 mg/kg) and S (1,360 mg/kg) as related to the other genotype groups. This group showed low grain concentrations of B (1.95 mg/kg), Se (0.10 mg/kg), Zn (39.2 mg/kg), Ca (327 mg/kg), Mn (20.0 mg/kg), Mo (1.75 mg/kg) and K (4,150 mg/kg) as compared to the other groups (Table 2).

Grain concentrations of B (2.10 mg/kg), Fe (38.5 mg/kg), Mg (1,290 mg/kg), Ca (408 mg/kg) and Mn (22.7 mg/kg) in landraces were high compared to the rest of the genotype groups. Low concentrations of Se (0.09 mg/kg), Zn (38.1 mg/kg), Mo (1.66 mg/kg) and K (4,000 mg/kg) were found in landraces as related to the rest of the groups (Table 2).

Cultivars were detected to have high grain concentrations of Ca (388 mg/kg), Mn (23.3 mg/kg), Mo (2.23 mg/kg), and low concentrations of B (1.59 mg/kg), Cu (4.49 mg/kg), Se (0.11 mg/kg), Fe (33.3 mg/kg), Mg (1,240 mg/kg), Zn (36.2 mg/kg), P (4,020 mg/kg) and S (1,230 mg/kg) as compared to the other genotype groups (Table 2).

### 3.2. Difference among Locations

Significant differences were found among the four locations in concentration of all minerals evaluated in the present paper (Table 2). Wheat grain samples from Alnarp showed significantly the highest concentrations of Se (0.15 mg/kg), Mg (1,300 mg/kg), Mo (2.19 mg/kg) and P (4,470 mg/kg) as compared to the other three locations. High concentration of K (4,180 mg/kg) was also found in the samples from Alnarp (Table 2).

Bohuslän wheat grain samples showed significantly the lowest B (1.45 mg/kg), Cu (3.79 mg/kg), Ca (330 mg/kg), P (3,300 mg/kg), S (988 mg/kg) and K (3,390 mg/kg) concentrations among all locations. Concentrations of Se (0.03 mg/kg), Mg (1,190 mg/kg), Zn (35.8 mg/kg) were also found to be low in grain samples from Bohuslän. Mn (41.2 mg/kg) in grains from Bohuslän was significantly higher than in those from Alnarp, Gotland and Uppsala (Table 2).

Grain concentrations of B (2.33 mg/kg) were significantly higher in samples from Gotland than in those from all other locations. Cu (5.34 mg/kg) and K (4,070 mg/kg) grain concentrations were also found high in samples from Gotland. Low concentrations of Se (0.04 mg/kg), Mg (1,220 mg/kg), Zn (36.2 mg/kg) and Mn (17.0 mg/kg) were noticed in grain samples from Gotland (Table 2). The lowest concentration of Fe (33.1 mg/kg) was found in grain samples from Gotland as compared to the other locations.

Among locations, Uppsala showed the samples with the highest Fe (49.6 mg/kg), Zn (43.4 mg/kg), Ca (411 mg/kg) and S (1,420 mg/kg) concentrations in comparison with samples from all other locations. Low grain concentrations of Mg (1,210 mg/kg) and Mn (17.0 mg/kg) were noticed in samples from Uppsala (Table 2). Mo (0.71 mg/kg) grain concentration was significantly the lowest in samples from Uppsala compared to Alnarp, Bohuslän and Gotland.

### 3.3. Difference between Spring and Winter Wheat

The data of the present study showed significant differences in mineral concentration between spring and winter wheat (Table 2). Spring wheat samples were observed to have significantly higher values for the concentrations of B (2.11 mg/kg), Cu (5.62 mg/kg), Fe (47.5 mg/kg), Zn (41.2 mg/kg), Ca (420 mg/kg), S (1,430 mg/kg) and K (4,160 mg/kg) as compared to winter wheat samples. Significantly higher grain concentrations of Mo (1.92 mg/kg) and P (4,260 mg/kg) were found in samples of winter wheat than in samples of spring wheat. The grain concentration of the minerals, Se, Mg and Mn did not differ significantly between spring and winter wheat samples (Table 2).

### 3.4. Correlation of Yield and Plant Height with Mineral Concentration

A negative correlation between yield and some of the evaluated minerals was found (Table 3). Thus, the grain concentrations of Cu (r = −0.58), Fe (r = −0.50), Mg (r = −0.40), Zn (r = −0.66), P (r = −0.53) and S (r = −0.40) were negatively correlated (P < 0.05) with yield (Table 3). Plant height was significantly positively correlated (P < 0.05) with grain concentrations of Cu (r = 0.28), Mg (r = 0.14), and P (r = 0.19) (Table 3).

### 3.5. Importance of Genotype Group and Location on Concentration of Different Minerals

Importance of genotype and location (measured by use of σ_g_^2^/σ_l_^2^) for grain concentration of various minerals was found to vary in relation to genotype group and mineral. For selections, location was found more important than genotype for concentration of all minerals except for the concentrations of Se and Mo (Table 4). For old cultivars, influence of the genotype was higher as compared to location for grain concentration of Fe, Mg, Mn and S. For the rest of the minerals (B, Cu, Se, Ca, Mo, P and K), location was found more important than genotype in old cultivars (Table 4).

For primitive wheat, genotype variation had higher influence for concentration of several of the minerals *i.e.*, B, Cu, Se, Mg, Zn, Ca, P, S and K as compared to location. Also, primitive wheat generally showed higher influences from genotype on concentration of minerals as compared to most of the other genotype groups (values above 1 indicated a higher number of minerals than the other genotype groups, and also generally higher values). With exception of concentrations of B, Se, and Mo in spelt wheat, all other evaluated minerals were more influenced by location than genotypes (Table 4). For landraces, influence of genotype was more pronounced than location for concentrations of Ca, Mn, Mo and K. For concentrations of B, Cu, Se, Fe, Mg, Zn, P and S location was more important than genotype among landraces. In cultivars, concentrations of Zn, Mo, P, S and K showed higher genotype influence than location (Table 4).

## 4. Discussion

The present study clearly showed the potential of producing wheat with exceptional high concentration of minerals by the use of specific genotypes in combination with organic cultivation (Table 5). Mean concentration of minerals from the organically produced whole grain flour in the present investigation was found to provide more than 70% of the daily intake of Cu, Se, Fe, Mg, Zn, Mn, Mo and P. This calculation was based on statistics from FAO [24] that the mean consumption of wheat flour is about 200 g per person per day. Furthermore, the calculations were based on the values for recommended intake for adults according to DGE [25] as shown in Table 5. However, the percentage of minerals in wheat grain was found to vary with different factors like genotype, environment, farming conditions *etc.*

More than three billion population of the world is facing deficiency of minerals. Minerals deficiency results in low working efficiency, high healthcare costs and increased rates of premature death [26]. Population of developing countries is especially at risk, because the people do not have enough money to buy mineral rich food such as meat, poultry, fish, fruits and vegetables. Fe and Zn are major deficient nutrients in the world [11]. The key factor for deficiency of Fe and Zn is the low concentrations in cereals [27]. Emphasis has been given on screening wheat genetic material for high concentrations of Fe and Zn within a large project led by the International Maize and Wheat Improvement Centre (CIMMYT) [28]. Thus, using specific genotypes in organic system might be the best tool to meet the minerals deficiency in humans.

In the present study high mean levels of most of the minerals were found as compared to previous studies (Table 5). The high mean levels were found despite the fact that a range of different types of wheat and wheat cultivars was included in this investigation. To our knowledge, all previous investigations in which genotypic variation in mineral concentration has been evaluated, except Ryan [14] have been carried out under inorganic conditions. Thus, the high concentration of minerals in the present study might be attributed to the organic farming system used and reflects the influence of soil supply of nutrients, efficiency of genotype uptake and yield. However, from previous studies [14,15] it might be concluded that an organic system by itself is not the clue of producing highly nutritious wheat in terms of mineral composition and concentration. Comparative studies have shown non significant differences in grain concentration of most minerals for wheat produced in organic and inorganic farming systems. In these studies, similar genotypes, mostly modern cultivars bred for inorganic systems, were grown under both systems [14,15]. However, the specific genotype can be a central factor to consider in organic farming systems. It is well known that the genotypes and production system interaction have a significant effect upon the performance of a genotype in a cropping system [29]. Genotypes that are bred for organic farming systems normally differ from those bred for inorganic systems in specific characters such as competitive ability with weeds, plant height, disease resistance, root system and nutrient use efficiency [30].

Considerable differences between grain concentrations of various minerals were not only found among genotypes, but also among genotype groups and type of wheat. The large variation among genotype, genotype groups and wheat type indicate the genetic potential that can be used to modify the minerals concentration in health promoting direction in wheat.

Generally, higher mineral concentration was observed for several of the investigated minerals in selections, primitive wheat, and old cultivars as compared to more modern wheat material, like e.g., cultivars and spelt wheat. However, for other minerals the relationship that more recent material showed lower concentrations of minerals could not be proven in the present investigation. Earlier investigations have indicated a negative correlation between more recent cultivars and amount of minerals [31]. This relationship has been attributed to a dilution effect of the minerals due to the increased yield of most recent cultivars [31]. Thus, in conclusion, the present study only partially agrees with previous results indicating decreasing mineral density in wheat over the last 160 years [31].

Our results showed that spring wheat generally contained higher concentrations of most minerals as compared to winter wheat. The decreased grain mineral concentration in winter wheat may be linked to a dilution effect because of their higher yield as compared to spring wheat. Also, a negative relationship was observed between the yield and mineral concentration in the present study. Findings of a negative relationship between yield and mineral concentration have been reported from other studies [12,32,33] although contrasting findings are present with a positive relationship between yield and mineral density [11]. Contrastingly, addition of fertilizer to get high yield in similar genotypes did not influence the concentration of minerals in grain [34]. Further, plant height of winter wheat (n = 128) were found to have a significant and positive correlation (P < 0.05) with Cu, Mg and P. Positive relationship of some minerals with plant height was also reported in earlier studies [12,31].

The amount of different minerals in the grain depends on the levels transported by roots during grain development and amount redistributed to the grain by vegetative tissue through the phloem [35]. Photosynthetic activity of vegetative tissue is an important factor in determining grain mineral concentration as well as the yield. Different wheat genotypes vary in photosynthesis and chlorophyll concentration [36]. Correlation between the chlorophyll and Fe concentration was reported by Dias *et al.* [37]. They found that higher chlorophyll content at final grain filling increased the amount of Fe in the grain [37]. Carbon dioxide enrichment was found to inhibit the assimilation of nitrate to organic nitrogen compounds. Low availability of nitrogen lead to reduced photosynthesis in wheat plant, also negatively affecting the grain quality e.g., mineral concentration [38].

From the present study, it appears that genetic differences are important in determination of mineral concentration of the wheat grain. Genetic difference for grain mineral concentration has also been reported from various varietal trials [9,11,12].

Ratio between genetic group and location variances (σ_g_^2^/σ_l_^2^) is helpful to measure the influences of genotype and location on the mineral composition of wheat. In this study, the genotype was found to have more impact on the mineral composition than location for primitive wheat. Thus, this strengthens the idea that primitive wheat genotypes can be used in organic farming system to enhance the mineral levels in wheat grain. In contrast, a previous study showed that relative influence of environment is higher than genotype for most of the wheat minerals [9].

Growing environment of different locations has an influence on mineral levels in grain [11]. In the present study, mineral concentration in the wheat grown in different locations was significantly different (P < 0.05). Variation in mineral elements of grain might be dependent on the soil characteristics of a particular location (Table 1). Cultivation in Alnarp and Gotland led to highest grain concentration of some of the minerals as compared to cultivation in Bohuslän and Uppsala. Increased organic matter percentage and pH of these locations might be the reason for higher mineral concentration. These findings are in accordance with previous studies which showed that high organic matter and pH favour the mineral concentration in the wheat grain [39,40]. Our material grown over six years did not show significant variation due to years. However, larger material grown over a number of years might show yearly variation as has been shown by [40].

In the present investigation, the mean mineral concentration in some genotypes were exceptionally high and were found to contribute near to 100% of recommended daily intake of most of the minerals. The wheat genotypes with the exceptionally high concentration of minerals under the used organic system came from all of the wheat genotype groups that were evaluated in the present investigation. Thus, these findings did not contribute to the idea that the old and more primitive wheat had higher potential to produce exceptionally high concentration of minerals in the grain under organic conditions. More important seemed the selection of the most optimal genotype for the production of high grain mineral concentration. For consumption of organic wheat, it is important to have in mind that concentration of minerals in the grain is dependent on which part of the grain that is used [41]. However, one idea of organic wheat grain consumption is to use whole grain flour products in order to receive high nutrition value in the food.

## 5. Conclusions

Wheat grain with high mineral nutritional value can be produced by using specific genotypes under organic cultivation. Mineral concentrations of organically grown wheat are higher than those seen in previous studies in inorganic systems. Thus, consumption of organically grown wheat flour could be a new strategy to alleviate human micronutrient undernutrition. Variation in mineral composition of different genotypes was found in this study, which can be used in further breeding to improve the nutritional quality of wheat grain.

By the choice of “the right” wheat genotypes together with suitable growing conditions *i.e.*, organic, it is possible to ensure almost daily requirements of minerals in the produced wheat. Examples of genotypes resulting in high mineral concentrations were Lant vete Gotland, *Triticum monococcum*, *T. dicoccum*, Lv. Dal, Olands Urval, *T. Polonicum*, APU and Schweiz.

## Supplementary Material

Supplementary Table 1

## Figures and Tables

**Table 1.** General characteristics of soil at different locations.

**Location**	**pH^a^**	**Organic matter (%)**	**clay (%)**	**P-AL b (mg/100g)**	**K-Al b (mg/100g)**	**FYM**	**Organic since**
**Alnarp**	7.0–7.8	3	25	10.5	10.2	Not applied	1992
**Bohuslän**	6.4–6.5	4	25	5.4	10.6	Applied	1995
**Gotland**	7.0–7.5	3	10	9.6	18.2	Applied	1987
**Uppsala**	6.0–6.2	5	50	9.9	46.2	Applied	1990

a from soil-water sample;

b P-Al and K-Al methods used [23].

**Table 2.** Mean concentration of minerals (mg/kg) among genotype groups, locations and wheat type for a total number of 493 wheat samples.

		**B**	**Cu**	**Se**	**Fe**	**Mg**	**Zn**	**Ca**	**Mn**	**Mo**	**P**	**S**	**K**
**Selections**	n = 32	1.59 c	5.27 b	0.18 a	35.8 abc	1,330 a	41.6 b	358 b	23.7 a	2.58 a	4,670 a	1,310 ab	4,050 b
**Old cultivars**	n = 191	1.90 bc	5.10 b	0.10 b	39.4 a	1,220 c	38.1 bc	390 a	24.2 a	1.53 b	3,890 d	1,260 bc	3,980 b
**Primitive**	n = 32	2.41 a	5.75 a	0.11 b	32.2 c	1,300 ab	45.6 a	383 ab	17.6 c	1.36 b	4,540 a	1,350 a	4,670 a
**Spelt**	n = 103	1.95 bc	5.50 ab	0.10 b	38.0 ab	1,280 abc	39.2 bc	327 c	20.0 bc	1.75 b	4,280 b	1,360 a	4,150 b
**Landraces**	n = 107	2.10 ab	5.33 b	0.09 b	38.5 ab	1,290 ab	38.1 bc	408 a	22.7 ab	1.66 b	4,130 bc	1,300 abc	4,000 b
**Cultivars**	n = 28	1.59 c	4.49 c	0.11 b	33.3 bc	1,240 bc	36.2 c	388 a	23.3 ab	2.23 a	4,020 dc	1,230 c	4,070 b
**Alnarp**	n = 278	1.84 b	5.29 b	0.15 a	38.4 b	1,300 a	39.9 b	382 b	24.2 b	2.19 a	4,470 a	1,320 b	4,180 a
**Bohuslän**	n = 29	1.45 c	3.79 c	0.03 c	38.2 b	1,190 b	35.8 c	330 c	41.2 a	1.33 b	3,300 d	988 c	3,390 c
**Gotland**	n = 141	2.33 a	5.34 ab	0.04 c	33.1 c	1,220 b	36.2 c	369 b	17.0 c	1.13 b	3,800 b	1,280 b	4,070 a
**Uppsala**	n = 45	1.79 b	5.66 a	0.08 b	49.6 a	1,210 b	43.4 a	411 a	17.0 c	0.71 c	3,500 c	1,420 a	3,890 b
**Spring wheata**	n = 176	2.11 a	5.62 a	0.10 a	47.5 a	1280 a	41.2 a	420 a	22.6 a	1.32 b	3870 b	1430 a	4160 a
**Winter wheata**	n = 317	1.86 b	5.05 b	0.10 a	32.5 b	1250 a	37.7 b	355 b	22.5 a	1.92 a	4260 a	1220 b	3920 b

Mean values followed by the same letter do not differ significantly from each other.

a Cultivation period for spring and winter wheat are April–August and September–August, respectively.

**Table 3.** Spearman rank correlation coefficients among various minerals related to yield and plant height.

	**Yield**	**Plant height**

**Number of samples**	n = 28	n = 128

**B**	−0.05	0.04
**Cu**	−0.58 **	0.28 ***
**Se**	−0.02	0.12
**Fe**	−0.50 **	0.08
**Mg**	−0.40 *	0.14 *
**Zn**	−0.66 ***	0.10
**Ca**	−0.13	−0.10
**Mn**	−0.26	0.04
**Mo**	0.19	−0.05
**P**	−0.53 **	0.19 **
**S**	−0.40 *	0.12
**K**	−0.27	−0.06

*, **, *** indicate significant at p < 0.05, 0.01, 0.005. Note: The yield was taken from 28 winter wheat genotypes grown in Bohuslän in bigger plots for reliable yield data. Plant height was measured for 128 winter wheat genotypes grown in Alnarp.

**Table 4.** Ratio of genetic to locations variance (σ_g_^2^/σ_l_^2^) for various mineral concentrations in different genotype groups.

**Groups**	**Selections**	**Old cultivars**	**Primitive**	**Spelt**	**Landraces**	**Cultivars**

**B**	0.20	0.69	2.35	1.41	0.96	0.62
**Cu**	0.38	0.91	2.45	0.81	0.87	0.95
**Se**	1.19	0.71	1.92	1.09	0.85	0.95
**Fe**	0.16	1.70	0.51	0.40	0.80	0.33
**Mg**	0.55	1.04	1.69	0.82	0.88	0.69
**Zn**	0.71	1.00	1.49	0.94	0.79	1.20
**Ca**	0.64	0.89	1.48	0.50	1.00	0.98
**Mn**	0.30	1.21	0.69	0.69	1.08	0.73
**Mo**	1.02	0.69	0.50	1.11	1.06	1.84
**P**	0.24	0.81	1.28	0.91	0.90	1.10
**S**	0.47	1.07	1.63	0.84	0.80	1.06
**K**	0.53	0.81	1.63	0.75	1.00	1.27

Value: <1 indicates more influence of location than genotype on mineral concentration; >1 indicates more influence of genotype than location on mineral concentration.

**Table 5.** Comparison of mineral concentration (mg/kg) in the present study with previous studies, recommended intake of minerals and percentage of recommended intake of minerals from flour consumption 200 g/person/day. This study was performed in organic system while rest of the studies, except Ryan *et al.* [14] were carried out under inorganic condition.

	**Present study**	**Spiegel et al., 2009 [40]**	**Zhao *et al.*, 2009** [12]	**Kirchmann*et al.*, 2009** [42]	**Fan et al., 2008 [31]**	**Ryan et al., 2004 [14]**	**Graham*et al.*, 1999** [11]	**Recommended intake (mg/day) according to DGE 2001** [25]	**Percentage of recommended intake from flour consumption 200 g/person/day**
**B**	1.96	0.69	n.a	n.a	n.a		2.3	1	39
**Cu**	5.26	3.9	n.a	3.51	4	3.3	n.a	1.5	70
**Se**	0.11	n.a	0.09	0.03	n.a	n.a	n.a	0.03–0.07	31–73
**Fe**	37.9	31	38.2	30.3	30.4	18	37.2	10	76
**Mg**	1,261	1,208	n.a	n.a	1,015	630	1,130	300–350	72–84
**Zn**	38.9	23.9	21.4	27.3	27.4	25	35.0	10	78
**Ca**	378	284	n.a	n.a	n.a	420	416	1,000	8
**Mn**	22.5	36.9	n.a	33.3	n.a	41	44.7	5	90
**Mo**	1.71	0.81	n.a	1.19	n.a	n.a	n.a	0.05–0.1	>100
**P**	4,124	3,293	n.a	n.a	n.a	2,800	3,380	700	>100
**S**	1,298	n.a	n.a	n.a	n.a	1,400	1,670	850–1,500	17–30
**K**	4,075	3,289	n.a	n.a	n.a	4,000	3,600	2,000	41

n.a indicates not analyzed.
